# Giant leiomyosarcoma of the inferior vena cava necessitating extended liver resection: A case report and review of the literature

**DOI:** 10.1093/jscr/rjab271

**Published:** 2021-06-22

**Authors:** Jacob Silverman, Niv Pencovich, Chen Mayer, Alexander Volkov, Rony Eshkenazi, Ido Nachmany

**Affiliations:** Department of General Surgery and Transplantation, Sheba Medical Center, Tel-Hashomer 52621, Faculty of Medicine, Tel-Aviv University, Israel; Department of General Surgery and Transplantation, Sheba Medical Center, Tel-Hashomer 52621, Faculty of Medicine, Tel-Aviv University, Israel; Institute of Pathology, Sheba Medical Center, Tel-Hashomer 52621, Faculty of Medicine, Tel-Aviv University, Israel; Institute of Pathology, Sheba Medical Center, Tel-Hashomer 52621, Faculty of Medicine, Tel-Aviv University, Israel; Department of General Surgery and Transplantation, Sheba Medical Center, Tel-Hashomer 52621, Faculty of Medicine, Tel-Aviv University, Israel; Department of General Surgery and Transplantation, Sheba Medical Center, Tel-Hashomer 52621, Faculty of Medicine, Tel-Aviv University, Israel

**Keywords:** Inferior vena cava, leiomyosarcoma, hepatectomy

## Abstract

Leiomyosarcoma of the inferior vena cava (IVC) is a rare malignant tumour of smooth muscle origin. It commonly presents with non-specific symptoms including abdominal pain, distention, and lower extremity edema. Surgical resection with macroscopically clear margins is the only potential curative treatment for the disease. Here we present the case of a previously healthy 38-year-old woman with a subacute one-month increase of a four-year slowly progressive right sided abdominal pain and back pain. Imaging revealed a 14.5x12x15cm mass in the right hepatic lobe causing mass effect on adjacent abdominal and retroperitoneal organs, and involving the retrohepatic IVC. En-bloc resection of the right hemi-liver, most of segment four, the caudate lobe, and approximately a 10 cm section of the retrohepatic IVC, along with IVC reconstruction, was performed. Histologic examination revealed the diagnosis of a high grade leiomyosarcoma.

## INTRODUCTION

Leiomyosarcoma is a malignant tumour of smooth muscle cell origin, which can occur in varied sites in the body, including the gastrointestinal tract, uterus, skin, and blood vessels [1]. 2% of leiomyosarcoma cases originate in blood vessels with half presenting in the IVC. Less than 400 IVC leiomyosarcoma cases have been reported to date, accounting for only 0.5% of all operated soft tissue leiomyosarcomas [2–4]. Surgical resection of the tumour with negative margins is the only intervention that has been demonstrated to improve survival [4]. Here we present a case of a massive IVC leiomyosarcoma necessitating an extended right hepatectomy with IVC resection and reconstruction.

## CASE PRESENTATION

**Figure 1:**
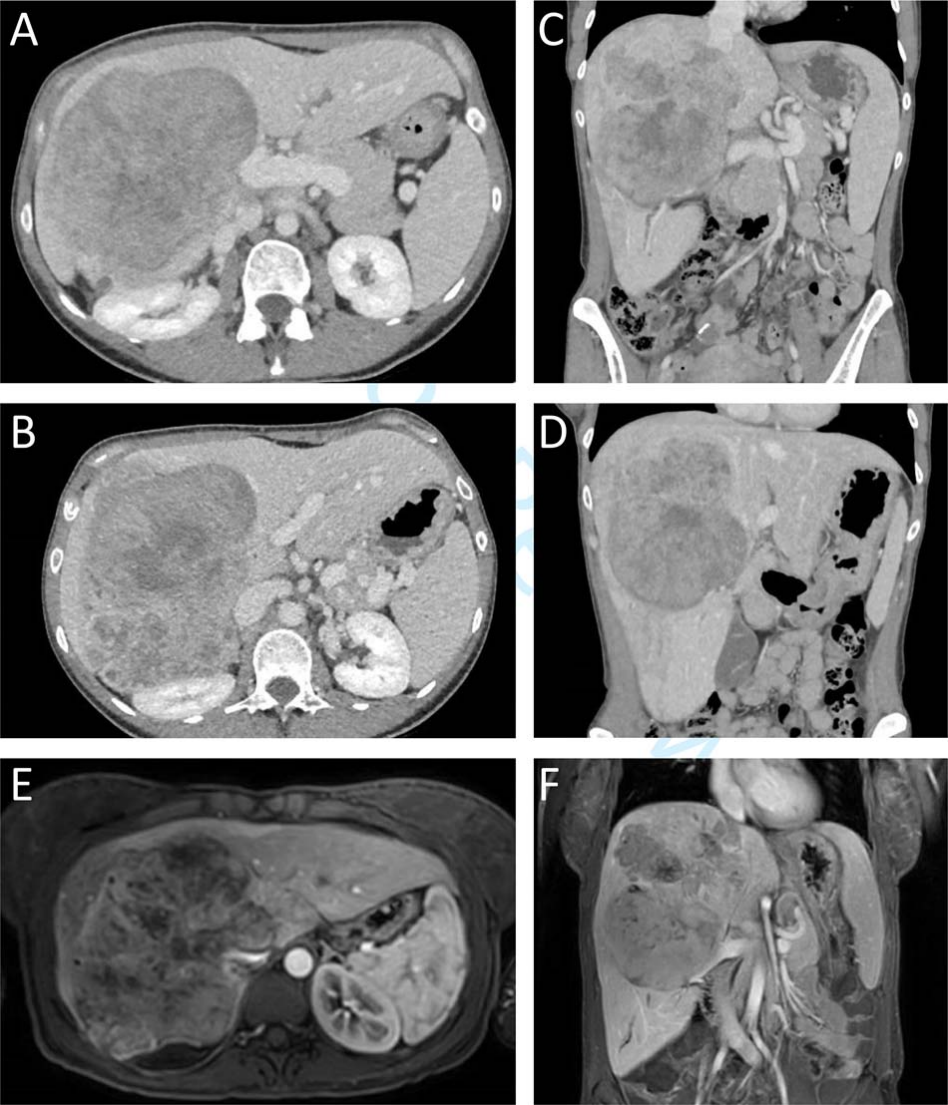
Preoperative CT and MR imaging. Axial (**A + B**) and coronal (**C + D**) CT images depicting a large mass replacing the right liver lobe, involving the retrohepratic IVC and compressing the right kidney. The portal bifurcation is free of tumour and the left portal vein is patent. (**E + F**) MR images depicting the large lesion occupying the right liver lobe and involving the retrohepatic IVC.

A previously healthy 38-year-old woman presented with a subacute one-month increase of a four-year slowly progressive right sided abdominal and back pain. Ten months prior to her visit she gave birth to a healthy child following an uneventful pregnancy. One month prior to her presentation, blood tests were taken as part of routine evaluation prior to in-vitro fertilization treatments that showed elevated serum hepatocellular enzymes. At the time of her admission an ultrasound was performed revealing an enlarged liver with a non-homogeneous echogenic structure in the right lobe, compressing the portal system. In light of these results and increasing pain she was referred to the emergency department. Upon her arrival her vital signs were normal. Physical examination revealed a large mass, slightly tender to palpation, in the right upper abdominal quadrant. Laboratory blood indices demonstrated a normal blood count, slight elevation of hepatocellular enzymes (ALT = 52, AST = 46) and cholestatic enzymes (GGT = 219, ALP = 435). Bilirubin levels were normal. The CA15.3 tumour marker was elevated (50.1 IU/ml). All other tumour markers including CEA, CA19–9, CA125, and AFP remained within normal limits. Abdominal computed tomography (CT) and magnetic resonance imaging demonstrated a 14.5x12x15cm mass in the right hepatic lobe causing mass effect on adjacent abdominal and retroperitoneal organs, and involving the retrohepatic IVC (Fig. 1). No signs of biliary obstruction were seen.

Since the lesion was resectable and no evidence of distant metastases was noticed upon imaging and lab indices, the patient was referred for surgical treatment without preoperative tissue diagnosis.

**Figure 2:**
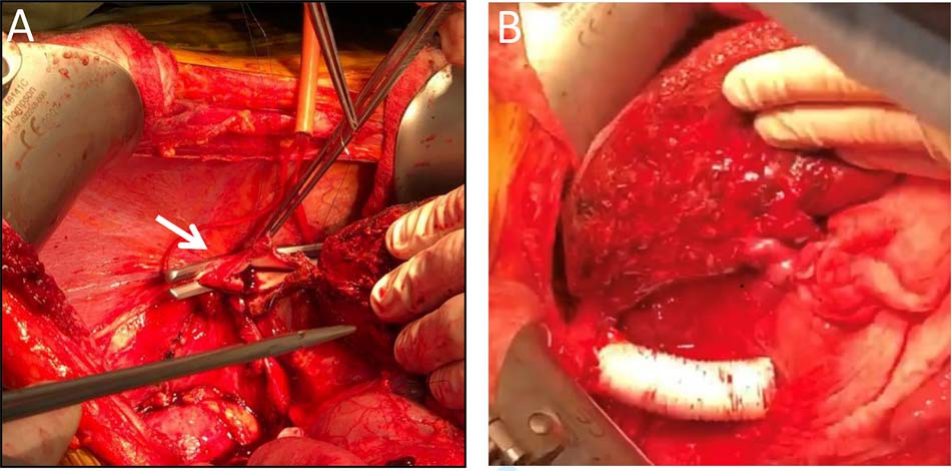
Intraoperative images. Intraoperative image depicting the remnant left lateral segment drained by the left hepatic vein into the IVC stump, marked by an arrow (**A**), and following IVC reconstruction with Gore-tex graft (**B**).

Surgical exploration via right subcostal incision with midline extension was performed. A giant tumour encompassing the entire right lobe including the middle hepatic vein as well as the caudate lobe and retrohepatic IVC was demonstrated. Femoral-Jugular venous–venous bypass was used during an en-bloc resection of the right hemi-liver, most of segment four, the caudate lobe, and approximately a 10 cm section of the retrohepatic IVC (Fig. 2). Reconstruction of the IVC was performed using a 20 mm Gore-tex graft (Fig. 2). A 20-minute bypass time was required. The surgery went uneventful with minimal blood loss. No blood products were administered during the operations.

**Figure 3:**
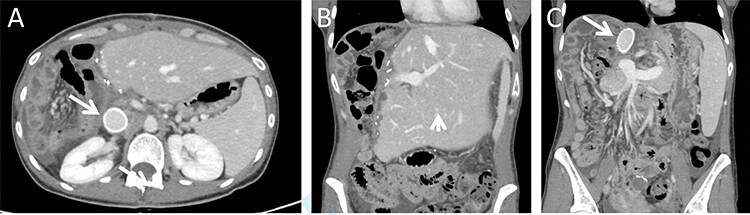
Postoperative CT imaging. (**A**) Axial CT image depicting patent IVC graft (arrow). (**B + C**) Coronal images depicting hypertrophied remnant liver (arrowhead), patent portal vein and IVC graft (arrow).

The immediate postoperative course was uneventful and the patient was discharged in good condition nine days following surgery. Twelve days after discharge the patient was readmitted due complaints of abdominal pain. Her vital signs, physical examination and laboratory blood indices were normal. A CT scan revealed a small amount of fluid in the abdominal cavity and thrombophlebitis of the left external iliac vein (Probably triggered by the cannula placed for venous–venous bypass). The remnant liver was markedly hypertrophied and the IVC Gore-tex graft was patent (Fig. 3).

**Figure 4:**
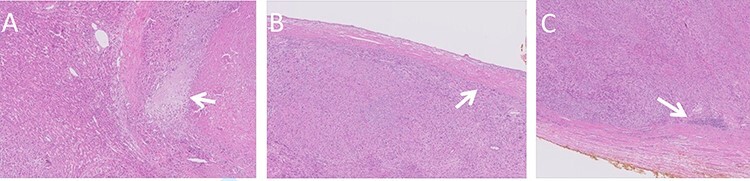
Histologic images of the resected lesion including IVC segment. (**A**) H&E staining, X20 magnification, demonstrating cells showing cytological atypia with areas of necrosis (arrow). (**B + C**) H&E staining, X20 magnifications, showing the tumour adjacent and originating from the IVC.

Macroscopic examination of the resected specimen observed a 1.92 kg liver lesion, measured 11x15x23cm. Attached blood vessels could not be separated from said lesion. Histologic examination revealed the diagnosis of a high grade leiomyosarcoma, with wide areas of necrosis, focally prominent cytological atypia and mitotic activity. Immunostains were diffusely positive for Calponin, Desmin, SMA and negative for Myogenin, CD34, S100, CAM5.2 and AE1/AE3. The tumour originates from a large venous type blood vessel – The IVC (Fig. 4). Inked resection margins were free of tumour.

## DISCUSSION

At present, complete surgical resection of the tumour is the only potentially curative treatment for IVC leiomyosarcoma [1, 2, 5]. The average survival of patients of patients treated medically without resection has been reported to be under three months [6]. Complete surgical resection of the tumour with negative margins has been reported to have five-year survival ranging between 31% to 66.7% [1, 4, 5, 7]. Due to the rarity of IVC leiomyosarcoma and paucity of large case series and clinical trials, the role of adjuvant chemotherapy and radiation remains unclear [2, 5, 6].

A surgical challenge involved in the resection of IVC leiomyosarcoma is reconstruction of the IVC. Reconstruction is postulated to reduce the risk of post-operative leg edema, as well as renal venous insufficiency. However, IVC ligation allows for the avoidance of long term anticoagulation. IVC ligation is considered for infra-renal lesions where the IVC is thrombosed on presentation, allowing for collateral circulation to have formed [4]. Tumours between the hepatic and renal veins necessitate IVC reconstruction to maintain renal venous drainage. Reconstructive options include a prosthetic or autologous graft, and primary repair. Maintaining IVC patency with prosthetic PTFE grafts is the most popular option [2]. Patency rates of up to 95% over a five-year period are described [8]. However, prosthetic graft reconstruction carries a risk of graft infection and reoperation due to bleeding as a result of long term anticoagulation [2, 5].

Under 40 cases of IVC leiomyosarcoma necessitating major liver resection were described to date, accounting for 20% of cases that have undergone resection [1, 2]. Multiple indications for hepatectomy have been shown. The presence of liver metastases has been an indication for simultaneous hepatectomy [9–12]. Liver resection has also been indicated due to the technical complexity and proximity of the tumour to the liver [12]. Tumour involvement of the hepatic vein confluence has a high likelihood of causing Budd-Chiari syndrome; as a result, it has been used as an indication for liver resection [13]. In the presented case, despite the lesion originating from the IVC, it appeared intrahepatic and encompassed the majority of the right liver, necessitating major liver resection for achieving an R0 resection.

In conclusion, current treatment for IVC leiomyosarcoma is surgical resection seeking macroscopically clear margins, with or without IVC reconstruction. The role of adjuvant therapy remains unclear, highlighting the need for further investigation of this unusual and complex disease.

## CONFLICT OF INTEREST STATEMENT

None declared.
